# The Liposuction-Induced Effects on Adiponectin and Selected Cytokines Are Not Affected by Exercise Training in Women

**DOI:** 10.1155/2014/315382

**Published:** 2014-01-02

**Authors:** Marina Yazigi Solis, Guilherme Giannini Artioli, Eduardo Montag, Vitor de Salles Painelli, Fábio Lopes Saito, Fernanda Rodrigues Lima, Hamilton Roschel, Bruno Gualano, Antonio Herbert Lancha Junior, Fabiana Braga Benatti

**Affiliations:** ^1^School of Medicine, University of Sao Paulo, Av. Dr. Eneas de Carvalho Aguiar, 255, 05403-000 Sao Paulo, SP, Brazil; ^2^School of Physical Education and Sport, University of Sao Paulo, Rua Professor Mello Moraes, 65, 05025-010 Sao Paulo, SP, Brazil

## Abstract

It has been suggested that the abrupt liposuction-induced decrease in adipose tissue could affect adipokine secretion pattern. We hypothesized that exercise training could positively impact adipokine metabolism following liposuction. The aim of this study was to investigate the effects of liposuction on inflammation-related adipokines in women who were either exercise-trained or remained sedentary after surgery. Thirty-six healthy normal-weight women underwent an abdominal liposuction and two months after surgery were randomly allocated into two groups: trained (TR, *n* = 18, four-month exercise program) and nontrained (NT, *n* = 18). Inflammation-related adipokine serum levels (TNF-**α**, IL-6, IL-10, and adiponectin) and abdominal and thigh subcutaneous adipose tissue (scAT) mRNA levels were assessed before (PRE) and six months after surgery (POST6). TNF-**α**, IL-6, and IL-10 serum levels were unchanged in both groups. In contrast, TNF-**α**, IL-6, and IL-10 mRNA levels in scAT were increased, whereas adiponectin scAT mRNA and serum levels were decreased at POST6 (*P* < 0.05, main effect for time). No changes were observed in mRNA levels of MCP-1, CD14, and CD68 in any of the groups. In conclusion, liposuction downregulates adiponectin scAT gene expression and serum levels and upregulates scAT gene expression of inflammation-related genes six months after surgery in normal-weight women, irrespective of exercise training.

## 1. Introduction

The adipose tissue is a complex and metabolically active tissue which secretes a variety of adipokines (e.g., adiponectin, TNF-*α*, IL-6, and IL-10) that play pivotal roles in metabolic regulation [1]. Thus, it has been suggested that the abrupt decrease in adipose tissue brought about by liposuction could affect adipokine secretion and, hence, the metabolic profile [2].

Previous studies on liposuction have shown somewhat conflicting results, with most reports showing no changes [2–6] while others demonstrate modest improvements [7–9] in one or more cardiometabolic risk factors, namely, insulin sensitivity, lipid profile, and proinflammatory cytokine levels. However, the lack of control for the subjects' physical activity level in these studies may be an important shortcoming as exercise may largely affect these parameters [10, 11]. For instance, we recently demonstrated [12] that a four-month exercise program was capable of improving insulin sensitivity in sedentary normal-weight women submitted to liposuction surgery whereas no change was observed in the nonexercised group. Additionally, most of these studies have evaluated obese subjects, even though liposuction is recommended as a cosmetic procedure for nonobese individuals [13].

Thus, the purpose of this study was to explore the effects of a small-volume abdominal liposuction on serum levels and adipose tissue gene expression of selected inflammation-related adipokines in normal-weight women who were either exercise-trained or not after surgery. We hypothesized that an exercise-training program could adjuvantly impact adipokine adipose tissue gene expression and blood levels following surgery.

## 2. Material and Methods

### 2.1. Experimental Design and Participants

A six-month randomized controlled trial was conducted (clinicaltrial.gov NCT01174485). This study was part of a clinical trial that aimed to comprehensively explore the effects of a small-volume abdominal liposuction associated with an exercise training program on body composition, energy expenditure, and cardiometabolic risk factors in women. The details of the study (e.g., sample's characteristics, surgery and biopsy procedures, exercise training program, and physical fitness and nutritional assessment) as well as its main findings (e.g., body fat distribution, energy expenditure, insulin sensitivity, lipid profile, and physical capacity) have been reported elsewhere [12].

Briefly, thirty-six normal-weight physically inactive women (20 to 35 years old; BMI = 23.1 ± 1.6 Kg/m^2^) were recruited. As previously reported [12], exclusion criteria included BMI over 30 Kg/m^2^; smoking; metabolic disorders such as glucose intolerance, diabetes, hypertension, thyroid dysfunction, and dyslipidemia; unstable body weight in the last six months prior to the commencement of the study; current use of medications including antidepressants, appetite suppressants, thyroid hormone medication, orlistat, topiramate, diuretics, anti-inflammatory, or antibiotics; any cardiovascular or musculoskeletal conditions that excluded exercise participation; and previous liposuction surgery. All of the subjects used oral contraceptives throughout the study. This study was approved by the local ethics committee. Before entering the study, all subjects provided written informed consent.

All participants underwent a small-volume abdominal liposuction surgery (mean of 1240.3 ± 363.6 mL harvested fat). Two months after surgery, subjects were randomly allocated into either a trained (TR, *n* = 18, four-month exercise training program) or a nontrained group (NT, *n* = 18). The exercise training program was performed three times per week and consisted of eight strength exercises for the major muscle groups (3 sets of 8–12 RM for each exercise) followed by 30–40 min of aerobic exercise on a treadmill at approximately 75% of the VO_2max_. As previously reported, adherence to the exercise program was 74.0 ± 13.2% and only the TR group showed a 12% increase in VO_2max_ (PRE versus POST6, *P* = 0.001) and a 38% increase in muscle strength as assessed by 1 RM leg press (TR versus NT, *P* = 0.001) after the intervention. Additionally, no changes in food intake were observed within or between groups throughout the study [12].

Prior to the intervention (PRE), before the beginning of the exercise program (i.e., two months after surgery(POST2)) and at the end of the study (POST6), subjects were assessed for serum levels of adiponectin, IL-6, TNF-*α*, and IL-10 using enzyme-linked immunosorbent assay kits (Quantikine HS; R&D Systems Ltd., Abingdon, UK) (Millipore, MA, USA).

Additionally, abdominal and thigh subcutaneous adipose tissue (scAT) samples were obtained by biopsies (as comprehensively described elsewhere) [12] and the gene expression of selected cytokines (adiponectin, IL-6, TNF-*α*, and IL-10) and macrophage-markers (CD14, CD68 and MCP-1) were assessed in a subsample (*n* = 6 per group). Reverse-transcription quantitative polymerase chain reaction (RT-qPCR) was performed as previously described [14] using the following primer sets: adiponectin, 5′-AGGCCGTGATGGCAGAGATG-3′, 5′-CTTCTCCAGGTTCTCCTTTCCTGC-3′; IL-6, 5′-AAAGAGGCACTGGCAGAAAA-3′, 5′-CATGCTACATTTGCCGAAGA-3′; IL-10, 5′-CAGCTGTTCTCCCCAGGAAA-3′, 5′-AGGGAGGCCTCTTCATTCAT-3′; TNF-*α*, 5′-CTGCCCCAATCCCTTTATT-3′, 5′-CCCAATTCTCTTTTTGAGCC-3′; MCP-1, 5′-CGACATCCTGGAACTGCCCTACC-3′, 5′-CACTGTGCCGCTCTCGTTCAC-3′; CD14, 5′-TAAAGGACTGCCAGCCAAGC-3′, 5′-AGCCAAGGCAGTTTGAGTCC-3′; CD68, 5′-GCTACATGGCGGTGGAGTACAA-3′, 5′-ATGATGAGAGGCAGCAAGATGG-3′; IPO8 (reference gene), 5′-CGAGAACGAGCTCAACCAGTCCT-3′, 5′-AGCTGCCTGTCGTACTGGGA-3′. Quantification cycle (Cq) and ΔCq values were calculated in every sample for each gene of interest as follows: Cq^gene of interest^−Cq^reference gene^, with IPO8 as the reference control gene. Relative changes in the expression level of the specific genes (Δ−ΔCq) were calculated by subtraction of the ΔCq at PRE (used as a calibrator) to the corresponding ΔCq at POST6. Finally, fold change was determined as 2^−Δ−ΔCq^. mRNA was purified from the adipose tissue biopsies using a commercially available kit following manufacture's recommendations (RNeasy Lipid Tissue Mini Kit, Qiagen). mRNA quantification and quality assessment were performed spectrophotometrically (NanoDrop 2000, Thermo Scientific) and integrity was verified through electrophoresis on a denaturing agarose gel. cDNA was synthesized from 1 *μ*g of mRNA using oligo-dT primers (Promega catalog number C1101) and M-MLV reverse transcriptase enzyme (Promega catalog number M170B). cDNA amplification and detection were conducted in a real-time thermal cycler (Rotor Gene Q, Qiagen) using the SYBR Green dye (Applied Biosystems, catalog number 4367659).

Finally, all of the POST6 assessments in the trained subjects were performed 60–72 hours after the last exercise training session.

### 2.2. Statistical Analysis

The dependent variables were compared using a mixed model for repeated measures assuming exercise training (TR and NT), time (PRE, POST2, and POST6), and adipose tissue depot (for the mRNA analyses only) as fixed factors and subjects as random factors (SAS 8.2, SAS Institute Inc., Cary, NC, USA). The significance level was set at *P* < 0.05. Data are presented as means ± SD.

## 3. Results

No between-group differences were observed at baseline for any of the parameters analyzed including age (TR: 26.6 ± 3.4 years; NT: 27.5 ± 4.8 years; *P* = 0.52, between-group difference).

TNF-*α* serum levels were undetectable in 18 subjects, IL-6 serum levels were undetectable in 1 subject, and IL-10 serum levels were undetectable in 2 subjects. Thus, sample sizes for TNF-*α*, IL-10, and IL-6 serum levels were TR: 7, 18, and 18; NT: 11, 17, and 16, respectively, for all times.

TNF-*α*, IL-6, and IL-10 serum levels were unchanged throughout the study in both groups. Adiponectin serum levels were unchanged at POST2 (*P* > 0.05) and markedly reduced at POST6 (*P* < 0.0001, 95% CI = 4.43 to 9.87, main effect for time) (Table 1).

**Figure 1:**
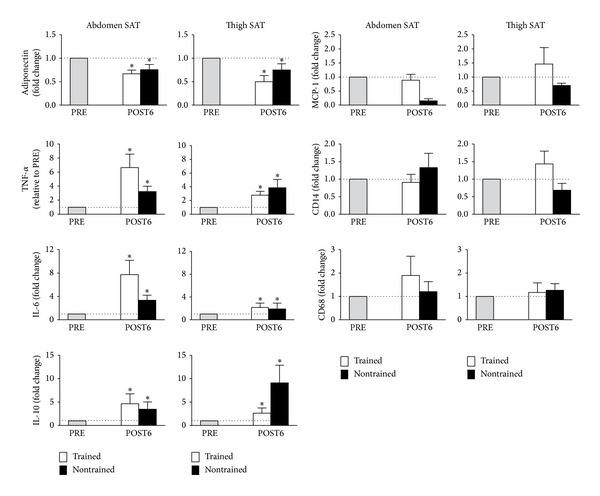
Subcutaneous abdominal and thigh gene expression of adiponectin, IL-6, IL-10, TNF-*α*, MCP-1, CD68, and e CD64. Data are expressed as means and standard deviation of fold change at POST6 with levels at PRE for both groups arbitrarily set to 1. ∗ indicates main effects for time (PRE versus POST6).

Abdominal scAT gene expression of TNF-*α*, IL-6, and IL-10 were significantly increased from PRE to POST6 (*P* = 0.003, 95% CI = −5.3 to −1.4; *P* = 0.0006, 95% CI = −10.7 to −3.9; and *P* = 0.03, 95% CI = −14.0 to −0.6, resp., main effect for time). A similar increase in TNF-*α*, IL-6, and IL-10 gene expression was observed in thigh scAT (*P* = 0.009, 95% CI = −3.9 to −0.7; *P* = 0.02, 95% CI = −3.4 to −0.4; *P* = 0.04, 95% CI = −9.0 to −0.2, resp.; main effect for time). In contrast, adiponectin gene expression was modestly but significantly decreased both in abdominal scAT (*P* = 0.05, 95% CI = −0.01 to 0.67; main effect for time) and in thigh scAT (*P* = 0.04, 95% CI = 0.01 to 0.78; main effect for time) (Figure 1). No changes were observed in mRNA levels of MCP-1, CD14, and CD68 in any of the groups. Additionally, there we no differences between abdominal and thigh scAT mRNA levels for any of the genes in either group throughout the study.

Finally, for a comprehensive overview of the results of the present study, Table 2 shows the previously published data [12] regarding the biochemical and anthropometric parameters, which were assessed at PRE, POST2, and POST6 in both groups.

## 4. Discussion

The main and novel findings of this study were twofold: (a) a liposuction surgery downregulates adiponectin scAT gene expression and blood levels and upregulates pro- and anti-inflammatory cytokine scAT gene expression; and (b) a structured exercise training program does not affect these responses.

Because of its remarkable secretory capacity, it has been suggested that scAT removal through liposuction could impact inflammation-related adipokine secretion and metabolic profile [2]. To the best of our knowledge, this is the first study to demonstrate a significant decrease in serum adiponectin levels, which was paralleled by a decreased adiponectin gene expression in scAT in normal-weight women after liposuction.

Decreased adiponectin levels have been consistently associated with impaired insulin signaling and insulin resistance and increased cardiovascular risk [15]. Interestingly, despite the significant decrease in adiponectin levels, our previously published data demonstrated no detrimental effects of liposuction on insulin sensitivity in the nontrained group. Furthermore, the exercise-trained group showed an improvement in this parameter further supporting a lack of association between the liposuction-induced responses in adiponectin and insulin sensitivity [12]. In fact, several studies have shown an uncoupling between changes in adiponectin levels and insulin sensitivity [16], indicating that factors other than adiponectin may regulate insulin action. Additionally, the fact that the subjects were not insulin resistant at baseline may partially explain these findings. Notwithstanding these observations, we cannot rule out the potential longer-term deleterious effects of liposuction on metabolism, especially when considering the pleiotropic beneficial effects of adiponectin [1].

The underlying mechanisms by which liposuction may downregulate adiponectin scAT gene expression and serum levels remain undisclosed. Nevertheless, the increase in scAT mRNA levels of TNF-*α* and IL-6 may have resulted in suppressed adiponectin expression in an autocrine manner, despite the concomitant increase in the anti-inflammatory cytokine IL-10 mRNA levels, [17, 18]. It is noteworthy that this increased scAT inflammatory milieu may not be accounted for an increase in macrophage infiltration as evidenced by the unchanged scAT mRNA levels of the macrophage-specific markers, but rather to an increase in adipocyte cytokine gene expression *per se*. Moreover, in our previously published paper [12], we showed data demonstrating that both adipocyte size and abdominal and thigh scAT mRNA expression of lipid metabolism-related genes (i.e., HSL, C/EBP-*α*, SREBP-c, LPL, and PPAR*γ*) remained unchanged throughout the study in both groups, with the exception of a decrease in thigh scAT gene expression of LPL and PPAR*γ* in the trained group only. Thus, it is unlikely that changes in adipocyte size or lipid metabolism could explain the current findings. Collectively our data points towards an inflammatory mechanism rather than a metabolic or an adipocyte function-related one. Importantly, previous studies have demonstrated a positive effect of dietary interventions on cytokines gene expression without any changes in adiponectin levels [19]. These data are hard to reconcile with those from the present study as they substantially differ in regard of their intervention protocols (i.e., dietary intervention versus liposuction surgery).

Importantly, although the exercise training program was effective in improving physical capacity and cardiometabolic risk factors in our sample as previously reported [12], exercise did not prevent the liposuction-induced impact on scAT cytokine expression profile. Previous studies have demonstrated improved circulating cytokine levels despite a lack of changes in scAT cytokine mRNA levels in response to an exercise program in overweight/obese subjects, suggesting a dissociation between serum levels and adipocyte gene expression of cytokines [20, 21]. This dissociation could be explained by posttranscriptional mechanisms and, most importantly, by the fact that immune cells, namely, monocytes, macrophages, dendritic cells, and T cells, may be the main accountable source for these cytokines' systemic levels [1].

This study was not without limitations. The follow-up period is rather short, thus warranting further long-term studies on the effects of decreased adiponectin levels on metabolism. Additionally, an exercise-only group would allow further insights on the effects of exercise *per se* on adipokines. The subjects in the present study were otherwise healthy at baseline, precluding the generalization of this data to other populations and the sample size for the gene expression analysis was relatively low. Finally, the gene expression analysis may not reflect adipose tissue actual synthesis and secretion of adipokines, that is, protein levels.

In conclusion, the results of this study indicate that a small-volume liposuction markedly downregulates the scAT expression and serum levels of adiponectin whereas it upregulates scAT expression of pro- and anti-inflammatory genes in normal-weight women, irrespective of an exercise training program. These findings suggest that a liposuction surgery may not be free of long-term metabolic effects in this population. Notably, although exercise training was incapable of counteracting these potential associated risks, it improved several cardiometabolic risk factors (data previously published) [12] and should be recommended following this procedure.

## Figures and Tables

**Table 1:** Effects of liposuction and exercise training on adipokine levels in normal-weight women.

Variable	PRE	POST2	POST6	Difference (CI 95%)	*P*
Adiponectin (*μ*g/dL)					
Trained (*n* = 18)	19.3 (7.3)^a^	17.5 (6.8)^a^	12.5 (4.1)^b^	7.6 (2.8 to 12.4)	0.0003
Nontrained (*n* = 18)	19.4 (8.0)^a^	18.1 (8.4)^a^	12.8 (3.9)^b^	6.7 (2.0 to 11.3)	0.001
TNF-*α* (pg/mL)					
Trained (*n* = 11)	0.64 (0.72)^a^	0.86 (0.99)^a^	0.52 (0.40)^a^	0.19 (−0.73 to 1.11)	0.99
Nontrained (*n* = 7)	0.83 (0.42)^a^	0.90 (0.58)^a^	1.22 (1.10)^a^	−0.36 (−1.50 to 0.78)	0.92
IL-6 (pg/mL)					
Trained (*n* = 18)	1.15 (0.90)^a^	1.38 (1.33)^a^	0.80 (0.34)^a^	0.30 (−0.56 to 1.14)	0.90
Nontrained (*n* = 17)	0.85 (0.55)^a^	1.29 (0.87)^a^	1.23 (0.88)^a^	−0.38 (−1.20 to 0.46)	0.77
IL-10 (pg/mL)					
Trained (*n* = 18)	1.78 (0.76)^a^	1.79 (0.73)^a^	1.69 (0.94)^a^	0.11 (−0.54 to 0.76)	0.99
Nontrained (*n* = 16)	1.55 (0.76)^a^	1.64 (0.47)^a^	1.42 (0.60)^a^	0.14 (−0.53 to 0.81)	0.98

Data are expressed as mean (SD), estimated mean of differences (confidence interval of 95%), and level of significance (*P*) between PRE versus POST6 (within-group comparisons; mixed model for repeated measures). Equal superscripted letters represent equal means and different superscripted letters represent statistically different means (*P* < 0.05, between- or within-group comparisons).

**Table 2:** Effects of liposuction combined with exercise training on body composition and blood parameters in adult women.

Variable	PRE	POST2	POST6	Diff (CI 95%)	*P*
Body weight (Kg)					
Trained (*n* = 18)	61.7 (5.4)^a^	60.8 (5.2)^b^	61.4 (5.7)^ab^	0.3 (−0.9 to 1.5)	0.98
Nontrained (*n* = 18)	59.7 (5.8)^a^	58.5 (6.1)^b^	58.9 (6.4)^ab^	0.9 (−0.4 to 2.1)	0.32
Fat mass (Kg)					
Trained (*n* = 18)	17.9 (3.0)^a^	16.7 (2.7)^b^	16.3 (2.8)^b^	1.6 (0.5 to 2.7)	0.001
Nontrained (*n* = 18)	17.6 (3.2)^a^	16.1 (3.1)^b^	16.6 (3.5)^ab^	0.9 (−0.2 to 1.9)	0.14
Lean mass (Kg)					
Trained (*n* = 18)	43.9 (3.7)^a^	44.2 (3.7)^a^	45.1 (3.8)^b^	−1.3 (−2.3 to −0.2)	0.008
Nontrained (*n* = 18)	42.3 (3.9)^a^	42.4 (4.1)^a^	42.2 (4.1)^a^	−0.1 (−1.1 to 1.0)	1.0
Abdominal scAT area (cm^2^)					
Trained (*n* = 18)	246 (42)^a^	166 (36)^b^	159 (30)^b^	87.2 (64.4 to 110.0)	0.0001
Nontrained (*n* = 18)	244 (52)^a^	170 (42)^b^	170 (49)^b^	73.2 (51.1 to 95.4)	0.0001
Abdominal VAT area (cm^2^)					
Trained (*n* = 18)	42.9 (10.2)^a^	41.2 (11.0)^a^	38.1 (9.1)^a^	4.5 (−0.4 to 9.4)	0.10
Nontrained (*n* = 18)	43.1 (14.9)^a^	42.5 (14.7)^a^	47.2 (14.2)^b^	−4.1 (−7.3 to −0.8)	0.01
Plasma leptin (ng/mL)					
Trained (*n* = 18)	27.2 (7.8)^a^	23.2 (7.5)^a^	21.6 (8.1)^a∗^	5.9 (−0.5 to 12.4)	0.08
Nontrained (*n* = 18)	27.2 (11.4)^a^	21.9 (10.1)^a^	28.4 (12.4)^a^	−1.2 (−7.2 to 4.9)	0.99
Plasma glucose (mg/dL)					
Trained (*n* = 18)	88.4 (8.8)^a^	86.5 (5.8)^a^	88.1 (7.4)^a^	0.3 (−5.4 to 6.0)	1.0
Nontrained (*n* = 18)	86.7 (8.9)^a^	87.0 (5.0)^a^	88.1 (6.6)^a^	−1.5 (−7.2 to 4.2)	0.99
Plasma insulin (mg/dL)					
Trained (*n* = 18)	7.7 (3.7)^a^	8.2 (6.6)^a^	7.1 (3.4)^a^	0.6 (−2.6 to 3.7)	0.99
Nontrained (*n* = 18)	8.7 (3.8)^a^	8.5 (3.3)^a^	8.2 (3.2)^a^	0.5 (−2.8 to 3.7)	0.99
AUC—glucose (mg/dL/min)					
Trained (*n* = 18)	13496 (2081)^a^	13507 (2390)^a^	12340 (2457)^a^	1156 (−677 to 2989)	0.40
Nontrained (*n* = 18)	12932 (1952)^a^	13698 (3191)^a^	13230 (3210)^a^	−298 (−2131 to 1534)	0.99
AUC—insulin (*μ*U/mL/min)					
Trained (*n* = 18)	6262 (2634)^a^	5213 (1655)^a^	4251 (1151)^b^	1738 (104 to 3371)	0.03
Nontrained (*n* = 18)	6725 (1966)^a^	6640 (2505)^a^	6496 (3013)^a^	482 (−1235 to 2199)	0.96

scAT: subcutaneous adipose tissue. VAT: visceral adipose tissue. AUC: area under the curve. Data are expressed as mean (SD), estimated mean of differences (confidence interval of 95%), and level of significance (*P*) between PRE versus POST6 (within-group comparisons; mixed model for repeated measures). Equal superscripted letters represent equal means and different superscripted letters represent statistically different means (*P* < 0.05, between- or within-group comparisons). *indicates *P* < 0.05 for single degree of freedom contrast analysis (between-group comparisons at POST6).
